# Liquid scintillation counting at the limit of detection in biogeosciences

**DOI:** 10.3389/fmicb.2023.1194848

**Published:** 2023-07-07

**Authors:** Florian Schubert, Jens Kallmeyer

**Affiliations:** GFZ German Research Center for Geosciences, Section Geomicrobiology, Potsdam, Germany

**Keywords:** background measurements, liquid scintillation, minimum detection limit, radioisotopes, biotic fringe

## Abstract

Liquid scintillation is widely used to quantify the activity of radioisotopes. We present an overview of the technique and its application to biogeosciences, particularly for turnover rate measurements. Microbial communities and their metabolism are notoriously difficult to analyze in low energy environments as biomass is exceedingly sparse and turnover rates low. Highly sensitive methods, such as liquid scintillation counting, are required to investigate low metabolic rates and conclusively differentiate them from the background noise of the respective analyzer. We conducted a series of experiments to explore the effects of luminescence, measurement time and temperature on scintillation measurements. Luminescence, the spontaneous emission of photons, disproportionally affects samples within the first few hours after sample preparation and can be minimized by following simple guidelines. Short measurement times will negatively affect liquid scintillation analysis or if background noise makes up a significant proportion of the detected events. Measurement temperature affected liquid scintillation analysis only when the temperature during the measurement reached approximately 30°C or higher, i.e. the liquid scintillation analyzer was placed in an environment without temperature control, but not in cases where chemicals were stored at elevated temperatures prior to measurement. Basic understanding on the functionality of a liquid scintillation analyzer and simple precautions prior to the measurement can significantly lower the minimum detection limit and therefore allow for determination of low turnover rates previously lost in the background noise.

## Introduction

The subsurface biosphere is the largest continuous ecosystem on the planet (Jørgensen and Boetius, 2007). The discovery of microorganisms hundreds or even thousands of meters below the ocean floor (Parkes et al., 1994; Inagaki et al., 2015) and in several kilometers depth in terrestrial habitats (Baker et al., 2003) illustrate that life in the subsurface is widely distributed.

Albeit extremely low microbial abundance and activity, the deep subsurface harbors ~15% of the world’s living biomass due to its sheer volume (Kallmeyer et al., 2012; Bar-On et al., 2018; Magnabosco et al., 2018). The subsurface biosphere is the intermediary between the mainly biologically controlled surface biosphere and purely abiotic geosphere and thereby plays a key role in important element cycles (Hinrichs and Inagaki, 2012).

A reoccurring theme of the investigation of the subsurface biosphere is exploration of the biotic fringe (Shock, 2000). Extremophilic microorganisms have been found in a variety of seemingly uninhabitable environments (Heuer et al., 2020; Beulig et al., 2022) and the known boundaries of life have been steadily extended over the last decades (Cowan, 2004). Particularly in deep subsurface environments, the often very low cell abundances and therefore low biomass at the biotic fringe hampers detection and quantification of microorganisms (Morono and Inagaki, 2016). Molecular biological techniques rely on extraction of sufficient amounts of biomolecules like DNA or RNA. Insufficient amounts of DNA lead to problems with low-level contamination from reagents and laboratory equipment, thus deteriorating the ratio between sample and contaminants. Given the severe limitations in available sample volume from deep subsurface environments, the total amounts of extractable biomolecules from such samples are often too small for even the most sensitive molecular biological analyses (Heuer et al., 2020). Highly sophisticated methods, such as cell separation via flow cytometry and cell sorting (Morono et al., 2013), may allow for cell quantification, but usually fall short to collect sufficient cells for phylogenetic analyses. Hence, no information about the composition of the microbial community can be extracted. This is a hurdle that often cannot be overcome, deeming culture-independent techniques like sequencing impossible (Heuer et al., 2020).

Incubations with radiotracer allow highly sensitive measurements of several quantitatively important carbon mineralization processes such as sulfate reduction, methanogenesis, fermentation, and anaerobic oxidation of methane (Jørgensen, 1982; Beulig et al., 2018; Jørgensen et al., 2019), that can give insight into microbial activity beyond the limits of biomolecular analysis (Heuer et al., 2020; Beulig et al., 2022). Short incubation times also allow for separate analysis of both directions of bi-directional reactions or steady states (Csala et al., 2005).

### Radioisotope Incubations

Radioisotope incubations are a standard technique for determination of catabolic and anabolic rates in sediments (Ivanov, 1956; Sorokin, 1962; Iversen and Blackburn, 1981; Smith and Klug, 1981). Major advantages of radioisotope incubations are their sensitivity and the fact that the concentration of the compound of interest does not significantly change, as the usually carrier-free tracer (i.e., without any non-radioactive components) has a very high specific activity and is only added in minute amounts. Microorganisms metabolize only a tiny fraction of the supplemented radioisotope and, in most cases, the radiolabeled product can still be analyzed. Additional sensitivity can be achieved by adding higher amounts of radiotracer to increase the chance of a labeled reagent being metabolized but comes with the burden of additional radioactive material.

Fossing (1995) outlined the major principles that have to be considered for radiotracer incubations:

The radiolabeled compound must be present only in trace amounts to keep the chemical and physical equilibrium of the system intact. The specific activity of the radioisotope [i.e., Bequerel (Bq) per mole of substrate] must be constant throughout the incubation. Therefore, a sufficiently short incubation time has to be chosen to keep turnover of the injected tracer <1%. As neither the concentration of the radioactive reagent nor the initial ratio of the reactants or other physicochemical parameters change significantly during the experiment, a constant turnover of the radiolabeled tracer throughout the entire incubation period can be assumed.

Jørgensen (2021) illustrates the significance of radioisotope techniques on the examples of sulfate reduction rate measurements by comparing the sensitivity of measuring changes in sulfate concentration via ion chromatography and ^35^S sulfate via liquid scintillation counting. The ^35^S method relies on conversion of ^35^S sulfate to ^35^S sulfide by sulfate reducing bacteria (SRB). Each sample is typically provided with 0.1–1 MBq ^35^S sulfate but only a small fraction is reduced by SRB. Liquid scintillation counters are able to detect less than 1 Bq of activity (Røy et al., 2014), i.e., only a millionth of the injected ^35^S, hence a millionth of the total sulfate pool. Ion chromatography has reproducibility of around 1%, so only concentration changes >1% can be safely detected. In conclusion, radiotracer techniques increase the sensitivity of measuring sulfate turnover about 10,000-fold.

Although turnover rate measurements do not provide any information about the composition of the microbial community, biological turnover of a specific (radiolabeled) compound can still be used to deduce the existence of a certain group of microorganisms even when microbial abundances are too low for molecular biological analyses (Heuer et al., 2020; Beulig et al., 2022). Additionally, while genomic data only provide information about metabolic potential but not about ongoing processes (these would require transcriptomic data), turnover of a radiolabeled substrate is unambiguous.

A combination of geochemical analyses and turnover rate measurements, e.g., anaerobic oxidation of methane, hydrogenase activity, methanogenesis, and sulfate reduction, can therefore provide novel insight into low biomass environments.

### Liquid scintillation counting

Liquid scintillation counting is a standard method for the quantification of low energy alpha- and beta-emitting radioisotopes. Liquid scintillation counting offers high counting efficiency for common isotopes involved in various chemical and biological cycles like ^3^H, ^14^C, ^32^P, ^35^S, and ^131^I and only requires relatively simple sample preparation. It is the method of choice for quantification of turnover and incorporation in a wide range of environments.

Liquid scintillation counting requires specific scintillation cocktails that consist of organic aromatic compounds and a suitable solvent. Decaying particles of the radioactive isotopes activate the aromatic solvent through electron excitation. The energy is then absorbed by the organic scintillator molecules, producing excited states of electrons and eventually emitting photons. The bursts of photons are detected by photomultiplier tubes (PMT) and converted into electric pulses. The amplitude of each electric pulse is hereby proportional to the decay energy. The electric pulses are then quantified and represent the detected signals given as output in the software of the liquid scintillation analyzer.

Due to the versatility of the liquid scintillation counting technique, it is used in a variety of fields such as medicine, meteorology, and physical sciences. Comprehensive literature about the Liquid scintillation counting method and functionality is available (Horrocks, 2012; Kobayashi, 2012; L’Annunziata, 2020; L’Annunziata et al., 2020) but almost exclusively focused on the previously mentioned main areas of application.

This publication aims to summarize relevant information for liquid scintillation counting in biogeosciences and to highlight important findings with respect to measurements near the absolute limit of detection.

### History and development of liquid scintillation counting

In the late 1920s and early 30s scintillation counting required manual detection of scintillation events with a microscope (Meyer et al., 1927; Krebs, 1955) but the methodology was eventually abandoned due to its labor intensity, subjective nature and difficulty as well as the development and rise of the Geiger-Müller counter.

Initial investigations of organic compounds and certain dissolved solutes as efficient scintillation sources by Kallmann were disrupted by the Second World War. After the war however, multiple publications (Broser and Kallmann, 1947; Herforth, 1948; Kallmann, 1950; Reynolds et al., 1950) presented the viability of organic solutions as scintillation liquids.

Most of the early liquid scintillation counters (LSC) were only equipped with a single photomultiplier tube (Horrocks, 1974) and therefore not able to detect low-energy beta emitters due to the high inherent background signal. The first commercially available LSC, the Tri-Carb 314, and Packard Instrument Company, was produced in 1953 (Temple, 2015). Since the inception of commercially available LSCs, innovations mainly focused on the reduction of the background via physical or electronic improvements (see the section “Background Reduction”).

### Commercial LS counters

The current market for LSC is shared by only a small number of manufacturers—PerkinElmer, Hidex, and Hitachi Aloka. Hitachi Alokas’ LB series is available almost exclusively in Japan and does not play a major role internationally. PerkinElmer’s Tri-Carb series is equipped with two main PMTs, facing the sample from opposite sides at one plane; some models have additional guard detectors with a PMT. The two-PMT design was also used in LSCs from Wallac Oy, Packard Bioscience, and Beckman. These companies discontinued their production, but their systems are still in use in many laboratories around the world. Hidex Scintillation Counters are a relatively recent addition to the market. Their two models (300/600SL) are both equipped with a triple PMT configuration, which enables determination of the triple-to-double-coincidence-ratio (TDCR). The TDCR method does not require an internal radiation source and allows to directly calculate counting efficiency. All of the above mentioned LSC allow for reliable determination of turnover rate measurements. Simple, portable analyzers like the Triathler LSC (Hidex) are also available for field measurements.

There are several factors that can influence the quantification of radioactivity via liquid scintillation counting. The most important ones are quenching and luminescence.

### Quenching

**Figure 1:**
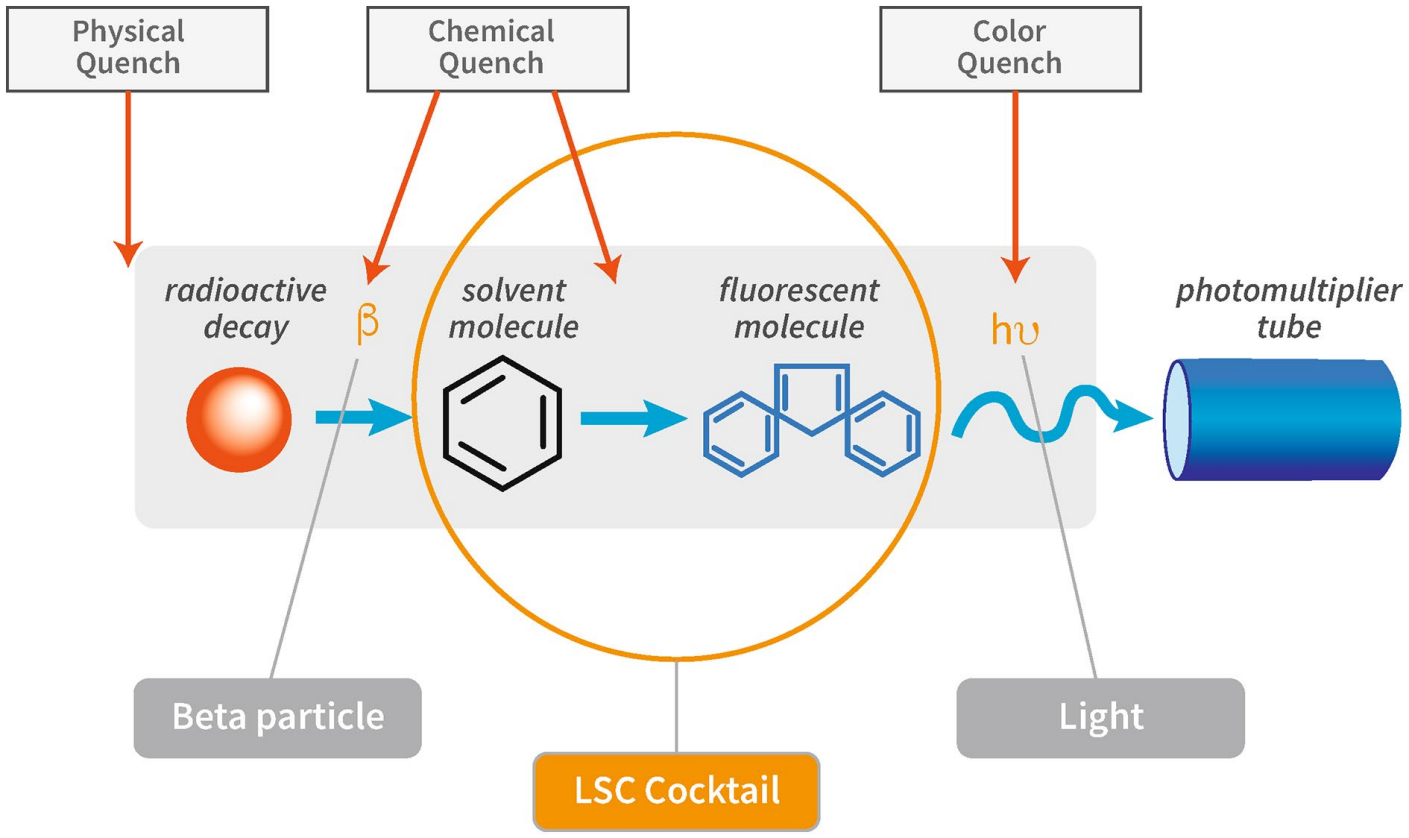
Schematic overview of the interference of quench on the different steps in the scintillation process. Modified after “PerkinElmer Introduction to Quench.”

Quenching is defined as incomplete transfer of the radioisotope’s decay energy to the PMT and results in an underestimation or loss of the amplitude of the signal. Different types of quench can occur, such as physical, chemical, color, and ionization quench (Figure 1).

Physical quench occurs when there is physical separation of the radioisotope and the scintillator, which can be a problem when working with solid scintillation techniques. It is of minor importance in the context of liquid scintillation counting as it can generally be avoided by proper homogenization of sample and scintillation cocktail. The properties of the cocktail should be checked to ensure its suitability for a given sample matrix in order to form a stable emulsion of sample and cocktail. Especially at very high or low pH values or high salt concentrations, the choice of the right LSC cocktail requires special consideration to suit the specific parameters. Also, the mixing ratio between cocktail and sample needs to be considered to ensure optimal counting efficiency (Røy et al., 2014).

Chemical quench describes the process of energy absorption between the radioisotope and the scintillator. The scintillation process is intercepted by chemical compounds in the sample or sample solution that scavenge excited molecules and produce heat instead of re-emitting the energy. Chemical quench represents the most frequent type of quench in liquid scintillation counting. Common chemical quenchers are Zn-ions, NaOH, ketones, organic acids, dissolved oxygen, aliphatic alkenes, and hydrocarbons (Cassette et al., 2000; Broda et al., 2007).

Color quench occurs when emitted photons are absorbed by coloration of particles or solutions before reaching the PMT. Examples for color quench include measurements of samples containing sediment or colored chemicals.

Ionization quench occurs when molecular damage is caused by a charged particle. The damage caused by the ionized molecule can either be temporary or permanent. Temporary molecule damage is characterized by high ionization density along the track of the ionizing particle. Permanent molecule damage is caused by long-term exposure of the scintillator to a high density of ionized molecules that negatively affect scintillation efficiency. The interaction between the liquid scintillator and a particle is a non-linear function of particle energy and non-linearity increases with stopping power of the particle (Broda et al., 2007). The stopping power is defined as loss of energy by ionizing radiation per unit of distance (Ashley, 1982). Photon emission of low-energy beta particles, such as those from ^3^H, or electrons will exhibit greater non-linearity, therefore cause higher ionization quench, than high-energy beta particles or electrons as interaction of the charged particles.

### Luminescence

One of the main contributors to the background (i.e., detection of events by the PMTs that are not associated with a sample’s radioactivity) is luminescence, which is a phenomenon caused by a low energetic single photon emission. Chemiluminescence and photoluminescence directly interfere with the assay of radioactive samples in scintillation cocktail and should be reduced and/or quantified for a reliable analysis.

Chemiluminescence is caused by chemical reactions between the scintillation cocktail and the chemical solution or substrate. Such chemical interactions can cause molecular excitation and light emission (L’Annunziata et al., 2020). The light is emitted when the excited molecule, in a state of an elevated energy level, decays into its ground state, the lowest possible energy level. Storage of the samples for a few hours can significantly reduce chemiluminescence.

Photoluminescence describes the effect of photon emission due to excitations by ultraviolet light (e.g., sun light) in the sample-scintillation cocktail mixture (L’Annunziata et al., 2020). Temporary storage for approximately 15 min in the dark prior to the LSC measurement should eliminate any photoluminescence. Samples can either be stored in dark environments or a pre-count delay in the LSC itself can be programmed. A subtype of photoluminescence, phosphorescence, can be encountered during wipe tests for contamination control. Detergents or cleaning agents are taken up by the wipe and then cause a reaction with the scintillation cocktail that can be characterized by a lower decay constant and longer duration.

### Triple-to-double coincidence rate

Liquid scintillation counters equipped with three instead of one or two PMTs allow for quantification of the Triple-to-Double Coincidence Rate (TDCR), which relies on a physical and statistical model of distribution and detection probability of scintillation photons. These models are the foundation for theoretical calculations of the counting efficiency, a critical parameter to evaluate the number of “true” counts. In TDCR systems, the PMTs are usually arranged in 120° angles to each other in one plane as it provides a geometrically optimal coverage of the measurement chamber. The underlying principle of TDCR is the comparison of counts that were measured by any combination of only two of the three PMTs and signals that were captured by all three PMTs and is calculated using the formula:


(1) $$TDCR=\frac{TripleCounts}{DoubleCounts+Triple\;Counts}$$

Triple-to-double-coincidence-ratio allows for the determination of a factor for quench correction, as common quenches such as color and chemical quench disproportionally affect the triple coincidences (Simpson and Meyer, 1994). The quench correction is a correlation between counting efficiency and measured TDCR and can routinely be applied to activity measurements of pure beta emitters irrespective of the states of quench. Counting efficiency can be determined by using external calculations (Simpson and Meyer, 1994) or is already included in the LSC-specific operating program. No external standard for the monitoring of quench level is needed and TDCR can be applied to both chemical and color quench, aqueous and organic samples as well as different scintillation cocktails and isotopes (L’Annunziata, 2020).

Triple-to-double-coincidence-ratio is also a viable analytical method for high-energy beta emitters, such as ^32^P, ^89^Sr, and ^90^Y by Čerenkov counting (Kossert, 2010), i.e., the detection of Čerenkov photons that are emitted by high-energy beta decay when moving in a dielectric and transparent medium (Čerenkov, 1937). An in-depth description of functionality and theoretical approaches of TDCR can be found in Broda (2003) and Broda et al. (2007).

### Background

The background is defined as the cumulative signal from external sources that are not associated with disintegrations in the sample. A variety of factors affects the background signal of a liquid scintillation counter, i.e., the inherent count rate: electrical noise of the analyzer, natural radioactivity in the instrument or in the vial material, ambient γ- and cosmic radiation, and the scintillation fluid. These interferences can be separated into a quenchable background and an unquenchable background. Quenchable background is caused by interactions of primarily cosmic radiation and natural radioactivity with the liquid scintillator solution whereas unquenchable background is caused by interactions outside the liquid scintillation cocktail (Horrocks, 1985).

Qualification and quantification of the counter background is of great significance for samples at or near the detection limit as an elevated or fluctuating background disproportionally affects such data. The same standards have to be applied for both counter background measurements and all actual samples, even those with count rates several orders of magnitudes above the background. For samples with high-count rates, the effect of the counter background on the data will be miniscule as the background only represents a tiny fraction of the signal but proper counter background assessment can help to identify potential contamination or carry-over of radioactivity in subsequent experiments.

A counter background measurement is carried out with a vial containing only scintillation cocktail and all other chemicals in the exact same ratio as in an actual sample but without any radionuclides. The identical ratio of chemicals will result in a comparable quench level between the counter background and sample measurements. The counter background then has to be measured for a sufficient length of time, optimally identical to the measurement time of the corresponding sample.

### Background reduction

Liquid scintillation counters are equipped with various tools to reduce the background. Those tools can be of physical nature like passive shielding or guard detectors or electric, e.g., pulse discrimination electronics. Commercially available liquid scintillation counters are using a combination of various techniques to automatically reduce background count rates. More details and supporting visuals about the various background reduction techniques can be found in L’Annunziata (2020) and L’Annunziata et al. (2020).

#### Passive shielding

A lead shielding is employed to reduce environmental gamma photons, cosmic muons, secondary X-rays, and thermal neutrons (L’Annunziata et al., 2020). The lead shield is composed of low residual activity lead and equipped with a layer of cadmium to shield against neutrons and can have an additional copper lining against stray magnetic fields (Kojola et al., 1984). The shielding can have a total weight of several 100 kg. Still, passive shielding does not fully absorb all high-energy photons and energetic cosmic particles (L’Annunziata et al., 2020).

#### Active guard detector

An active guard detector includes additional PMTs that either surround the sample or are placed very close to it, but lack an optic path for scintillation photons from the sample to enter the tube. Any event simultaneously detected by the PMT of the active guard and the sample detector will be rejected, as it represents an external signal not associated with the disintegration of the sample. Photon detection by only the internal PMTs will be attributed to a decay inside the measurement chamber and counted as a sample-derived radioactive decay or luminosity, depending on the energy profile and duration of the pulse. An active guard is the most efficient way to reduce background and filters much of environmental gamma radiation and 99% of soft cosmic components (L’Annunziata, 2020; L’Annunziata et al., 2020).

#### Pulse discrimination electronics

Pulse shape analysis (PSA) and pulse amplitude comparison (PAC) can be applied to carry out pulse discrimination analysis.

The LSC detects different signals, namely nuclear decay or background events, which do not share the exact same properties. The shapes of the pulses of the detected signals are determined by their energy and decay time. Nuclear decays appear with a strong, prompt pulse and rapid decay whereas background events produce weaker pulses with a longer decay time. The photons emitted by the fast decay, from excited single states with paired electrons, have a duration of typically 2–8 ns while the delayed pulse, from annihilation of more stable triplet states with unpaired electrons, can persist for several hundred nanoseconds (L’Annunziata, 2020). Pulse shape analysis can be used to differentiate between a true nuclear decay and a background event due to their unique pulse shape. Optimization of the pulse shape analysis is specific to vial material and sample-cocktail chemistry (L’Annunziata et al., 2020). PSA is most effective for alpha counting but has also been used for background discrimination (Kaihola et al., 1991) and separation of background events from beta decay (L’Annunziata, 2020).

The ratio of pulse amplitudes detected by different PMTs can be analyzed via a pulse amplitude comparison. When PMTs detect photons generated by nuclear decay within the scintillation cocktail, the pulse amplitudes at the different PMTs will be very similar; hence, the ratio of pulse amplitudes between the PMTs will be close to 1. Cosmic radiation or naturally occurring decays in the material of the vial (e.g., in glass vials) will result in a higher amplitude on one PMT and a weaker amplitude at the other PMT(s) causing a dissimilar signal and hence a ratio deviating from 1. Pulse amplitude comparison should not be applied to measure low-energy beta-emitters or Čerenkov radiation as only a small number of photons is generated (L’Annunziata et al., 2020).

#### Physical background reduction (vial, cocktails)

The physical background of the sample can be reduced by choosing appropriate vials and scintillation cocktail. Vials can have an inherent background due to the material containing radioactive elements, e.g., ^40^K in glass vials. While specific low ^40^K glass vials are available, plastic vials provide multiple advantages including a lower background, as they are made from ^14^C-dead hydrocarbons and should not contain any natural radioactive compounds. Plastic vials require a scintillation cocktail that will not dissolve the vials (i.e., cocktails not containing solvents such as benzene or toluene). Additionally, plastic vials may be affected by buildup of static charge, which leads to elevated count rates (L’Annunziata et al., 2020). Specific antistatic vials or a built-in deionizer in the LSC are efficient ways to circumvent the static charge build-up.

As mentioned above, it is important to use the same chemical composition, i.e., sample-cocktail ratio, for background and sample analysis. An optimal ratio should be determined for every specific LSC cocktail-sample mixture.

#### Stable counting environment

The liquid scintillation counter should be operated in a controlled environment as parameters, such as temperature, humidity, and sunlight can affect the background. Optimal temperature windows for LSC operations and scintillation cocktails are provided by the respective supplier, and deviations from those recommendations can significantly affect background levels and variation within the background. As mentioned previously, direct exposure of the samples to sunlight will lead to elevated rates of photoluminescence. Hence, environments without direct sunlight should be prioritized for the placement of the LSC.

### Motivation

Increasing interest in and accessibility to deep subsurface sediment or other samples from environments near the biotic-abiotic fringe led to a growing need for measurements of turnover or incorporation rates close to the limit of detection. However, such measurements demand a deep understanding of the parameters that affect the limit of detection. A low limit of detection is the fundamental requirement to reliably detect turnover rates in samples from low biomass or low activity sites. Therefore, we investigate different parameters such as temperature and measurement time to monitor their effect on the limit of detection to provide recommendations and to improve detection of very small amounts of radioactivity as often encountered in low turnover rate analyses.

## Materials and methods

### Scintillation counting

Liquid scintillation analysis was conducted with a Hidex 600 SL LSC. The LSC is equipped with a triple PMT configuration that allows recording double coincidences (CPM2), counts that are detected only at two of the three PMTs, and triple coincidences (CPM3), which are simultaneously detected at all three PMTs, within a pre-defined coincidence time of 35 ns. Measuring CPM2 and CPM3 allows for the determination of the triple-to-double-coincidence ratio. Additionally, the LSC is equipped with an additional active guard PMT underneath the counting chamber to detect ambient radiation.

All background samples were prepared with a 7/8 mL sample/cocktail mixture; in case of counter background measurements, the sample consists of 7 mL 5% zinc acetate (ZnAc) without any added radioactivity. The composition of the counter background sample was chosen because the ZnAc is used as final trap in the cold chromium distillation (Kallmeyer et al., 2004), a widely used technique to measure sulfate reduction. This method uses ZnAc to capture the microbially produced H_2_^35^S in the form of Zn^35^S. All sample-cocktail mixtures were vortexed for 10 s to ensure homogeneity. Prior to analysis sample vials were cleaned with microfiber tissues moistened with ethanol to remove potential contaminants on the outside walls. The standard measurement time was 10 min, but dedicated measurements with a measurement time of 1, 2, and 60 min were also carried out.

Experiments that investigated luminosity and/or the effect of measurement time were carried out with the vial repeat option that allows measuring the same sample vial multiple times in a row without removing it from the measurement chamber, with a delay of a few seconds between each measurement.

### Luminosity and counter background

For the assessment of the decline of luminosity with time and its effect on the background, a freshly prepared background sample containing 7 mL 5% ZnAc and 8 mL scintillation cocktail was put into the scintillation counter immediately after preparation and cleaning of the outside of the vial with ethanol. These background samples were measured consecutively for 200 times with a measurement time of 1 and 2 min and 100 times with a measurement time of 10 and 60 min, respectively. For each experiment, new counter background samples were freshly prepared—a total of 12 samples, one for each combination of the four measurement times and three scintillation cocktails. The assessment of luminosity and the counter background was carried out with the following scintillation cocktails with the same measurement time and number of measurements: ROTISZINT Eco Plus (Carl Roth, Germany), AquaLight+ (Hidex, Finland), and Ultima GOLD XR (PerkinElmer, United States). We will refer to these cocktails by the following abbreviations: ROTI, AL+, and UG, respectively.

### Measurement temperature and storage temperature

We stored our scintillation cocktails in a dark environment at room temperature as per manufacturers’ guidelines. In order to investigate the temperature optimum for LSC measurements, we conducted a series of tests. Eight counter background samples were measured at each of the temperature steps.

In a first experiment, we increased the measurement temperature from 10°C to over 35°C to simulate the approximate annual temperature range in a room without proper temperature control. For this test, we used ROTISZINT Eco Plus and Ultima GOLD XR scintillation cocktail.

In a second experiment, potential chemical effects of elevated storage temperatures on the scintillation cocktail were tested by heating background samples in an incubator to various temperatures between 25 and 60°C. We used the same cocktails, ROTI and UG, as in the previous experiment and prepared eight replicates of each cocktail. A triplicate of background samples kept at 20°C was used as a reference.

## Results

### Assessment of the counter background

#### Luminosity

**Figure 2:**
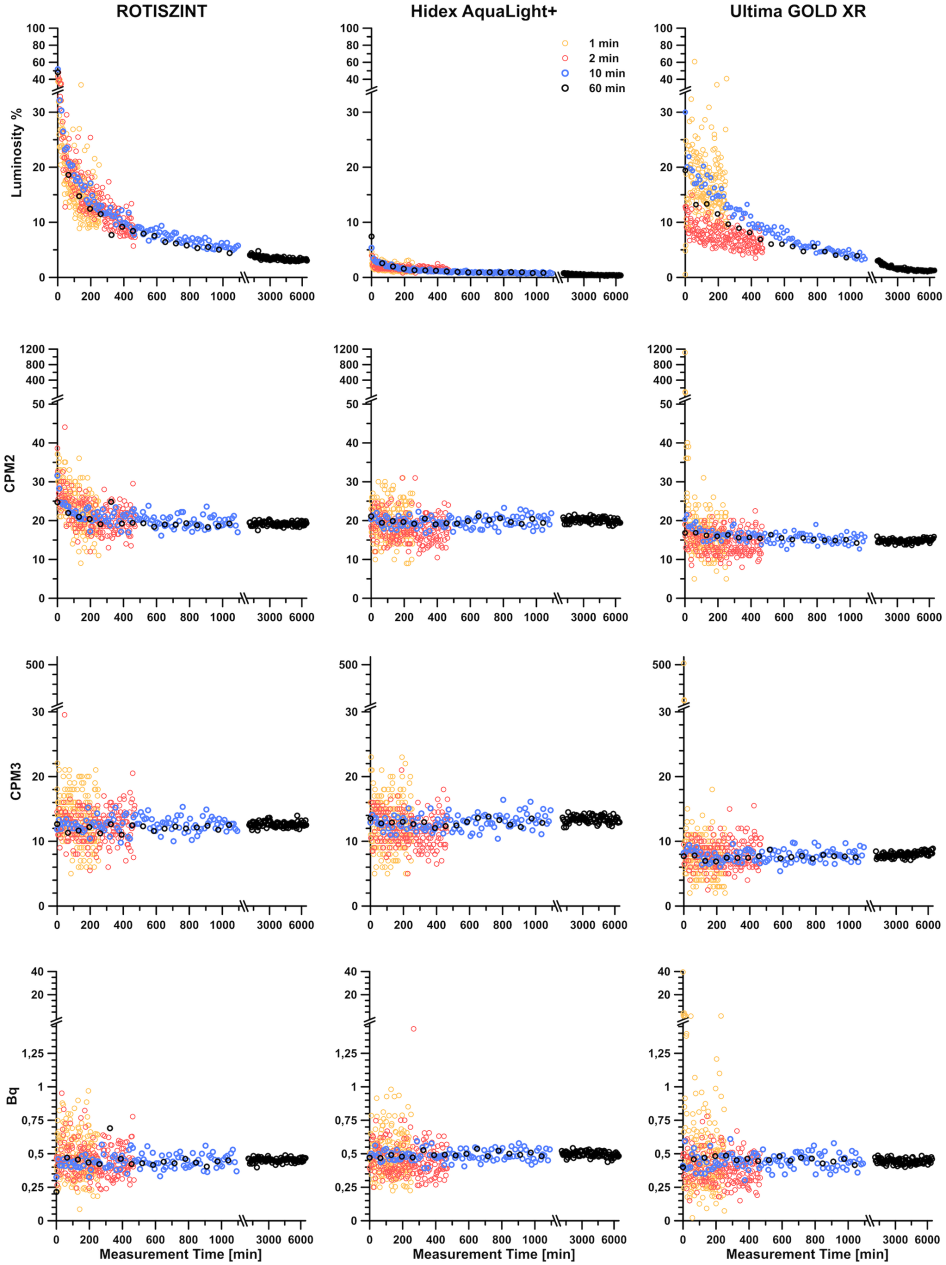
Contribution of luminosity on detected counts and related double detections (CMP2), triple detections (CPM3), and Becquerel values over a measurement period of up to 4  days, with varying measurement times and different scintillation cocktails. Counter background measurements were carried out with 1, 2, 10, and 60 min of measurement time. A time series for each background measurement was established by repeating the analysis of the sample for 200 times (for experiment with a measurement time of 1 and 2 min) and 100 times (for experiments with a measurement time of 10 and 60 min). Three different scintillation cocktails (ROTISZINT, Carl Roth; AquaLight+, Hidex; and Ultima GOLD XR, and Perkin Elmer) were investigated with the above mentioned measurement times.

We regularly observed noticeable differences in luminosity in our standard background measurements that we run at the beginning, middle, and end of each sample analysis. This difference correlated with time passed since the start of the measurement; with high luminosity in the samples measured early in the sequence and significantly lower luminosity at the end. For all measurements (Figure 2), irrespective of scintillation cocktail or measurement time per sample an initial peak for luminosity was observed in the very first measurement repetitions followed by a asymptotic decline in luminosity over the next few hours, which eventually resulted in a plateau. While the general trend is similar between all measurements, significant differences were observed between the various measurement times and scintillation cocktails.

In terms of luminosity, the AL+ scintillation cocktail yielded low luminosity values from the start and also quickly established a plateau with low and consistent luminosity values. The cocktails ROTI and UG show a compareable luminosity profile. Both have initial luminosity values of >20% that decline below 10% after *ca.* 10 h and experience a constant decline even after a measurement time of more than 4 days. The UG cocktail had a slightly less pronounced initial peak and lower minimum values than the ROTI cocktail. In terms of luminosity, the 1-min measurement experiment with UG cocktail showed significant scatter of almost 30% even after almost 5 h of measurement time.

#### Counter background

Independent of the scintillation cocktail, the measured counter background in the various experiment revealed almost identical trends. Comparing the background experiments with 1, 2, 10, and 60 min of measurement time, the scatter of measured background activity is declining with increasing measurement time (Tables 1–3), i.e., while data of the 1-min measurement time experiments show a range of >20 CPM2 or > 0.75 Bq, data of the 60-min measurement time experiment are confined within ~5 CPM2 or < 0.1 Bq. For measurement times of 2 min and longer all cocktails performed equally realiable.

**Table 1:** Performance of selected scintillation cocktails at various measurement times.

[CPM2]	1 min	2 min	10 min	60 min
ROTISZINT	23.48 ± 5.38	21.59 ± 4.28	20.60 ± 2.30	19.32 ± 0.99
AquaLight+	19.70 ± 4.56	18.07 ± 3.33	19.75 ± 1.42	20.04 ± 0.64
Ultima Gold XR	23.07 ± 77.95	14.58 ± 2.87	15.96 ± 1.60	14.94 ± 0.63

Performance is presented by CPM2 (events counted by two of the three PMTs) of the counter background values and respective standard deviation.

**Table 2:** Performance of selected scintillation cocktails at various measurement times.

[CPM3]	1 min	2 min	10 min	60 min
ROTISZINT	13.49 ± 3.56 (±3.67)	12.49 ± 2.63 (± 2.50)	12.62 ± 1.14 (±1.12)	12.52 ± 0.49 (±0.45)
AquaLight+	12.92 ± 3.76 (±3.59)	11.82 ± 2.42 (±2.43)	12.97 ± 1.17 (±1.14)	13.32 ± 0.55 (±0.47)
Ultima Gold XR	10.31 ± 36.25 (±3.21)	8.03 ± 1.97 (±2.00)	7.80 ± 0.91 (±0.88)	7.90 ± 0.42 (±0.36)

Performance is presented by CPM3 (events counted by all three PMTs) of the counter background values and respective standard deviation. Values in parentheses are calculated values based on the Poisson distrubution. Except for the 1-min measurement of Ultima Gold XR, the calculated and measured values are in good agreement.

**Table 3:** Counter background of selected scintillation cocktails at various measurement times.

[Bq]	1 min	2 min	10 min	60 min
ROTISZINT	0.48 ± 0.16 (0.96)	0.45 ± 0.12 (0.81)	0.44 ± 0.05 (0.59)	0.45 ± 0.04 (0.57)
AquaLight+	0.49 ± 0.16 (0.97)	0.45 ± 0.14 (0.87)	0.49 ± 0.04 (0.61)	0.50 ± 0.02 (0.56)
Ultima Gold XR	0.73 ± 2.80 (9.31)	0.39 ± 0.11 (0.72)	0.45 ± 0.06 (0.63)	0.45 ± 0.02 (0.51)

Performance is presented by the average Becquerel of the counter background values and respective standard deviation. The resulting minimum detection limit (MDL), calculated as counter (background plus three times standard deviation is given in parentheses).

##### CPM2 vs. CPM3

Initial measurements of CPM2 seem to follow the trends of luminosity for approximately 300 min, although the asymptotic decline is less pronounced. This effect is especially visible for shorter measurement times. After the first 300 min, double detections (Figure 2) follow a linear trend around 20 CPM2 for the ROTI and AL+ and 15 cpm for UG scintillation cocktails (Table 1). Overall, UG reveals the lowest values for CPM2 and CPM3 while AL+ has lowest luminosity of all cocktails. However, UG is also heavily affected by outliers, especially during the first hour, for measurement times of 1 min that result in extremely elevated values for CPM2 and standard deviation (Table 1).

With the exception of the 1-min measurements, each of the cocktails shows relatively small variability in their average cpm values, but their standard deviation decreases drastically with increasing measurement time (Table 1). While for a measurement time of 1 min AL+ appears to perform slightly better than the other two cocktails, UG outperforms the other cocktails at longer measurement times, revealing both lowest CPM2 values and smallest standard deviation.

Triple detections (CPM3) behave similar to the CPM2 but detected values are significantly lower. The CPM3 values do not seem to be influenced by luminosity as the initial spike in luminosity that can be observed within the first few hours of measurements is not visible. Additionally, CPM3 values are extremely consistent from the first to the last measurement. In comparison, the first 100 min of measurements of CPM2 in the presence of high luminosity (e.g., for ROTI) are vastly different from the last 100 min of measurements (Figure 2). This effect, much like in the luminosity profiles, is more pronounced for shorter measurement times and the ROTI and UG scintillation cocktail. ROTI and AL+ show an average of around 12 CPM3 (Table 2) while the average of the UG cocktail is around 8 CPM3. Again, measurement time only has a minimal influence on the CPM3 with slightly elevated values for the 1-min measurements while standard deviation decreases with increased measurement time. The 1-min measurements of the UG cocktail show highly increased values during the first 2 h of measurements, which leads to a significantly higher standard deviation (Table 2). Between CPM2 and CPM3, CPM3 has a lower average cpm value and lower standard deviation (Tables 1 2).

##### Becquerel values

The calculated Becquerel values incorporate features of the three parameters above. For all cocktails and measurement times, the Bq values average around 0.45 Bq (Table 3) with only one major excursion in the UG 1-min measurement time data set. This excursion is mainly influenced by the generally poor performance of the cocktail at 1-min measurement time, especially with regard to its luminosity. The standard deviation visibly decreases with measurement time and is reduced to 25–12.5% of its original values between the 1- and 60-min measurement experiments.

### Impact of measurement temperature on background measurements

**Figure 3:**
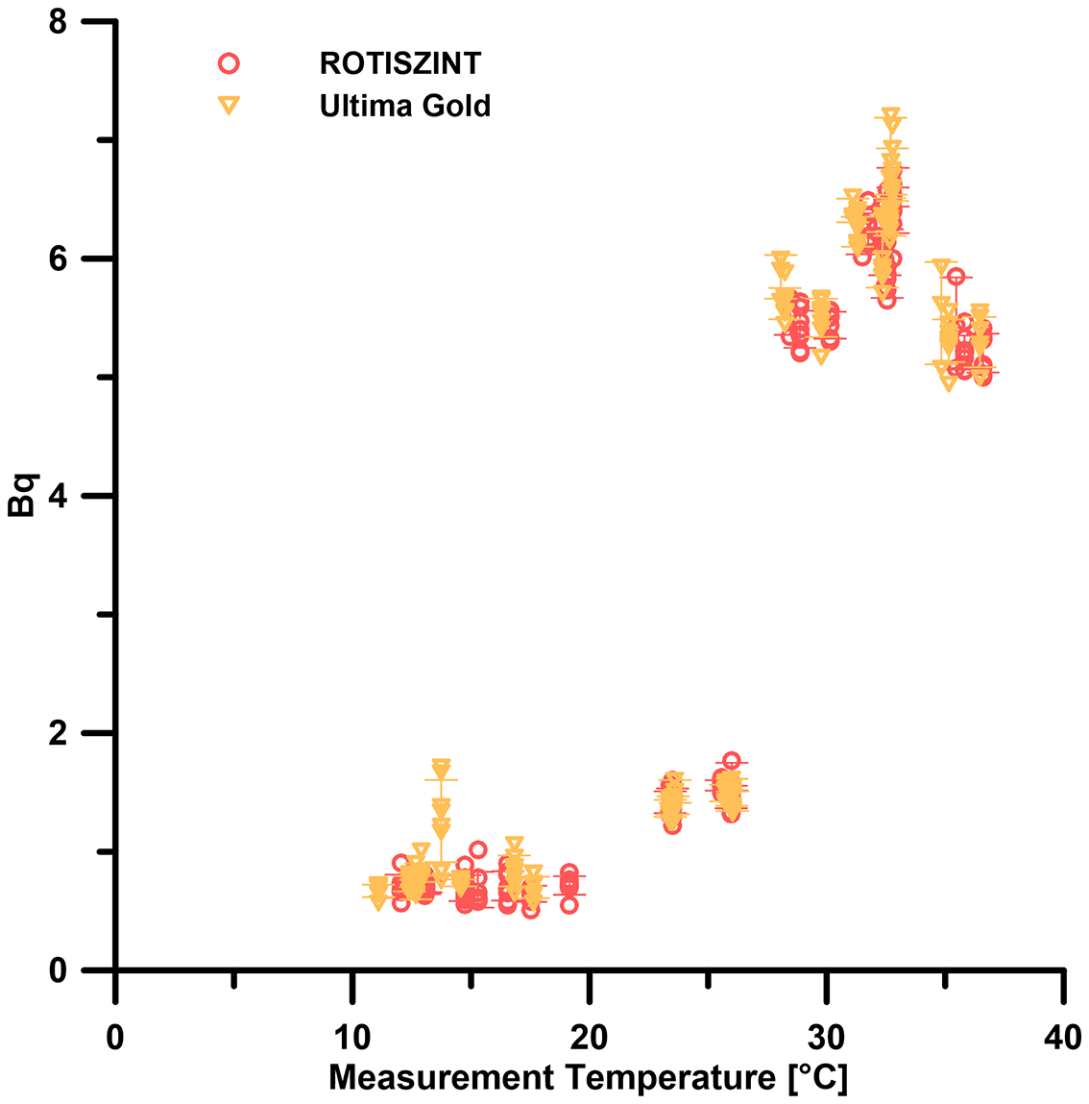
Effect of measurement temperature on the background measurements of different scintillation cocktails. Error bars indicate one standard deviation.

Our data show that at measurement temperatures of 10–20°C, all background measurements remain consistently below 1 Bq with only one minor excursions at 14°C with six out of eight vials having an activity >1 Bq for the UG scintillation cocktail (Figure 3). Around 25°C, the background measurements for both scintillation cocktails increase to mean values around 1.5 Bq. As soon as the measurement temperature reached 28°C, we observed a drastic jump to 5–7 Bq for both cocktails. Values remained at this level up to the highest measurement temperature of 36.5°C. Except for the minor excursion in the low temperature range, the response to measurement temperature was consistent for both scintillation cocktails analyzed. The cocktails performed reliably at 10–20°C and started to jump to highly elevated values around the 30°C mark.

### Effect of storage temperature on background measurements

**Figure 4:**
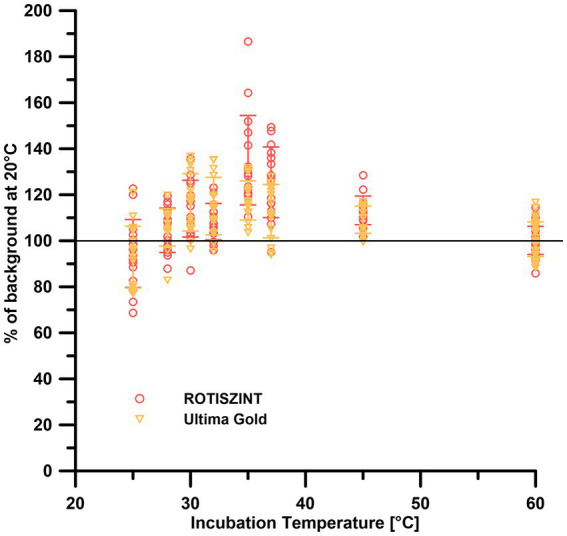
Effect of storage temperature on background measurements of different scintillation cocktails. Error bars indicate one standard deviation.

The data of the temperature-dependent background measurements, irrespective of the incubation temperature, are generally similar to the 20°C reference background (Figure 4)—around ±20%. The UG scintillation cocktail performed consistent throughout the temperature range of 20–60°C with a maximum range of 80–140% of the 20°C reference measurements. While the ROTI cocktail mostly lies in the range of 70–130% of the reference values, elevated values of up to 190% of the 20°C reference can be observed at 35–36°C.

## Discussion

### Luminosity

L’Annunziata et al. (2020) mention that photoluminescence will generally disappear if the sample is stored in a dark environment for 15 min and chemiluminescence is expected to last for an approximate 4–6 h. Luminosity will affect samples disproportionately within the first few hours and generally drops below 10% after 8–10 h. However, only after 4 days a plateau is reached. The AL+ scintillation cocktail with its initial low luminosity appears to be a preferable choice for measurements where luminosity is of concern. Our experimental setup does not allow for the distinction between photo- and chemiluminescence, but the high initial peak likely reflects a superimposed signal of both prevalent luminescence components that quickly changes to an asymptotic decline once the influence of photoluminescence wears off. This effect is pronounced for shorter measurement times, especially the 1-min measurements, due to low numbers of photon detections per measurement. Interestingly, the 1-min measurements of the UG cocktail also show some strong negative outliers within these first few minutes.

The effect of luminosity can be mitigated by computational means that modern LSC are equipped with, but additional consistency can be achieved by simply storing sample vials in the dark for at least 1 day, preferably 3 or 4 days prior to the measurement, or by utilizing a scintillation cocktail that naturally shows low luminosity. For background measurements, a measurement time of at least 10 min should be targeted as it represents a good compromise between statistical significance and duration (Figure 2). If measurement time is of no concern, a longer measurement time of, e.g., 60 min will improve performance, although the improvement is not dramatic, as illustrated by the changes in background counts and standard deviation at different measurement times (Table 2).

In general, a consistent measurement protocol and precautions such as sufficient time between sample preparation and measurement allows for better comparability of the samples and avoids potential discrepancies between different measurements caused by changes in luminescence over time.

### CPM in double and triple PMT setups

With the increasing availability and popularity of commercial LSCs with a triple PMT configuration, a distinction between cpm values of different types of liquid scintillation counting has to be made. On a double PMT setup, both PMTs (PMT A and PMT B) oppose each other and are arranged at a 180° angle inside the measurement chamber. Photon detection on both PMTs (AB) will equate to one count of sample-associated radioactive decay. In comparison, the PMTs in a triple PMT setup (PMT A, PMT B, and PMT C) are orientated at 120° angles and sample-associated decay requires the detection on all three PMTS (ABC).

Due to the different geometries and number of PMTs, the detection limits with regard to cpm values may differ between machines, with a lower limit of detection achieved by the geometrically more complex triple PMT setups. Next to the advantage of having an extra PMT to improve photon detection, the TDCR method also allows for statistical modeling of expected double and triple coincidences and compares expected detections to the ratio of measured CPM2 and CPM3. In turn, the comparison of double detections will result in higher CPM2 values in the triple PMT setup as a double detection can result from multiple PMT combinations (i.e., AB, AC, and BC). TDCR in a triple PMT setup will include the detection on only two of its three PMTs and perform a quench correction that provides the opportunity to calculate the respective Bq value on the basis of the ratio of double detections (CPM2), triple detections (CPM3), and luminosity.

The formula for our luminosity corrected TDCR values is the following:


(2) $$TDCR_{corr}=\frac{CPM2}{CPM3-Lumi}$$

Where Lumi represents all detected decays not associated with radioactive decay of the sample which are determined via probabilistic distribution of double and triple counts. On the basis of the luminosity-corrected TDCR, the luminosity-corrected Bq can be calculated using the formula:


(3) Bqcorr=CPM2TDCR260

The use of cpm, even between different PMT setups, has the advantage of more direct comparability, although cpm values are likely skewed toward lower numbers for the triple PMT setups. The utilization of the TDCR method and calculated luminosity-corrected Bq values are only possible with triple PMT LSC as the statistical foundation for calculating Bq values requires both CPM2 and CPM3.

### Measurement time

Accuracy of the measurement increases with measurement time as the measurement time is correlated to the number of detected signals, i.e., a 10-fold increase of measurement time approximately results in a 10-fold increase in detected events (Røy et al., 2014). A visualization of this statement can be seen for our 1- and 2-min experiment (Tables 1–4) that consistently performed much worse in terms of consistency than the 10- and 60-min measurements. Radioactive decay are random, independent events that occur at a fixed mean rate, i.e., decays follow Poisson distribution. Thus, the rate of decay of the targeted radioisotope and ambient and cosmic radiation has an underlying variability. These statistical fluctuations within the background signal or radioactive decay result in minute variations for any specific time frame. Therefore, longer measurement times are preferred to reduce variations between measurements as they cover a larger number of expected decays. These fluctuations can be reduced significantly by targeting a measurement time of at least 10 min. Throughout all measured scintillation cocktails, we found a decrease in standard deviation between the 1- and 10-min background measurements to less than 30% of their initial cpm and Bq values. For our experiments and throughout all tested scintillation cocktails, experiments of 60-min measurement time yielded the best results but come with the burden of prolonged time investment especially for large numbers of samples. A measurement time of 10 min provides a good compromise between low and consistent measurements and short measurement time.

Further, we can compare the observed (standard deviation of Table 2) to the expected standard deviation (Table 2) of the counter background to evaluate if variability within the background fully matches the Poisson process. The standard variation of the Poisson process is calculated with $\sqrt{\lambda }*t^{-1}\;$ where λ is the total amount of counts over the respective measurement interval and *t* is the measurement time. The comparison shows that both observed and expected value are extremely close (**Δ** ≈ <0.1). Thus, the counter background is Poisson distributed and the standard deviation is inherited by the Poisson process. The only exception and significant deviation of the observed and expected standard deviation is the aforementioned 1 min measurement of the UG scintillation cocktail that shows an observed variance of 10 times the expected value. However, it is unclear what exactly attributes to the stark difference between the observed and expected value.

A compromise between statistical significance and duration of a measurement has to be made, as data sets can be comprised of several hundreds of individual measurements. Short measurement times will significantly increase the impact of background noise (Figure 2) while long measurement times will be statistically sounder but more time consuming. Røy et al. (2014) mention that the number of counts will reach a point of diminishing returns, a reduction in statistical significance per time spent. Generally, a counting time of 10 min is accepted as a good compromise between statistical significance and time and is used by many studies (Kallmeyer et al., 2004; Beulig et al., 2022; Nagakura et al., 2022). However, many publications do not provide any information about their measurement time (Boetius et al., 2000; Böning et al., 2004; Kallmeyer and Boetius, 2004; Treude et al., 2005; Røy et al., 2014). In samples with medium to high activity, the number of detected decay events is several orders of magnitude higher than in low turnover samples and therefore well above the minimum detection limit set by the blank measurements. Measurement protocols that exceed the 10-min counting time can be applied to increase the number of counts and therefore decrease variance of the background noise (Røy et al., 2014). Other studies that operate at the limit of detection utilize longer measurement times (30 min; Glombitza et al., 2016). Our results show that an extended measurement time will have an improved detection limit by 1–2 cpm or 0.05 Bq if measurement time is increased from 10 to 60 min (Table 4).

Kallmeyer et al. (2004) investigated the effect of measurement time on the minimum detection limit and proposed that 10 min is a sufficient amount of time and further increase will not lower the detection limit any further. Furthermore, Røy et al. (2014) presented a statistical analysis focusing on the number of detected decay events and concluded that a higher number of detected events leads to statistically better results. However, the improvement of the results is affected by diminishing returns and eventually leads to impractical long measurement times. Our results (Tables 2 3) support the concept of diminishing returns on measurement time, although still show a benefit of measurement times >10 min for determination of the minimum detection limit. Nevertheless, a significantly further extension of measurement time beyond 60-min will not lead to a markedly improved minimum detection limit due to diminishing returns. Additionally, hour-long measurement times can only be applied to small sample sets due to the time constraints. The effect of the diminishing returns is illustrated in Table 2. Comparing the standard deviation of the 2-, 10-, and 60-min measurements, i.e., increasing measurement time 5 or 30 times, leads to a reduction of standard deviation by ~50 or 75%, respectively. The standard deviation for CPM3 drops from 2 to 2.6 cpm of the 2-min measurements to approximately 0.5 cpm for the 60-min measurement. The diminishing returns are even more obvious for Bq measurements (Table 3) as the 60-min measurement already reaches a standard deviation of almost zero (~0.02 Bq). Extensive measurement times would result in a large enough number of expected decays to no longer have any variation between measurements but also would be impractically long.

Figure 2 illustrates the effect of insufficient counting times. The figure shows consecutive background measurements, but while counting times of 2 min or higher show an exponential decline of luminosity over time, a counting time of 1 min does not show this trend. Moreover, the 1-min data show significant scatter of more than three times the standard deviation of the 10-min measurement. This scatter can also be seen in the corresponding calculated activity values (Figure 2; Bq).

The effects of different measurement times on the minimum detection limit can be shown by using, e.g., the CPM3 values for AL+ (Table 2). The minimum detection limit (Kaiser, 1970) is defined as:


(4) $$MDL=b+3\times \sigma_{b}$$

Where *b* is the average value of all counter background measurements and σ_b_ is the standard deviation of the blank signal. A factor of 3 is chosen to reflect the approximate 95% confidence interval of the counter background. This calculation of the MDL is used as an approximation due to its simplicity. A statistically correct treatment based on the true distribution of counter background that is used in Røy et al. (2014) to present detection near the absolute limit of detection on the example of sulfate reduction is available in Brüchle (2003).

The MDL (4) for the UG cocktail will be 13.94 cpm (2 min), 10.53 cpm (10 min), and 9.16 cpm (60 min), thus result in a difference of >4 cpm or > 33% between 2- and 60-min measurement times. The effect of counting times on microbial turnover rate experiments is more directly visible and easier to understand when using Becquerel values (Table 3). For the UG cocktail, the MDLs (4) for the 2, 10, and 60 min measurements are 0.72, 0.63, and 0.51 Bq, respectively. An increase in measurement time for the UG scintillation cocktail from 2 to 10 or from 10 to 60 min results in a reduction of the detection limit by approximately one 10th of a Bq. Recent studies that target the biotic-abiotic fringe use tracer activities of up to 5 MBq per sample for quantification of sulfate reduction rates in the sub-picomolar range (Glombitza et al., 2016; Beulig et al., 2022; Nagakura et al., 2022). With these low microbial turnover activities a reduction of a single cpm or fraction of a Becquerel can be decisive on whether a measurement is above or below MDL and therefore potentially change the conclusions derived from such a dataset. In the following, we show how small changes in the counter background affect the MDL, using a typical sample for quantification of sulfate reduction rate measurements as an example. Sulfate reduction rates are calculated according to the following formula:


(5) $$SRR=\left[SO_{4}\right]\times P_{SED}\times \frac{a_{TRIS}}{a_{TOT}}\times t^{-1}\times 1.06\times 10^{6}$$

Where SRR is the sulfate reduction rate in pmol cm^−3^ day^−1^; [SO_4_] is the sulfate concentration in the porewater of the sediment in mmol L^−1^; P_SED_ is the porosity of the sediment in mL porewater cm^−3^ sediment; a_TRIS_ is the radioactivity of the total reduced inorganic sulfur, a_TOT_ the total radioactivity used; t the incubation time in days; 1.06 the correction factor of the isotopic fractionation (Jørgensen and Fenchel, 1974); and 10^6^ the factor for the change of units from mmol L^−1^ to pmol cm^−3^.

Assuming [SO_4_] of 10 mM, P_SED_ of 0.6, a total radioactivity used of 5 MBq, incubation time of 10 days and a SRR of 0.1 pmol cm^−3^ day^−1^, the amount of radioactivity in the TRIS fraction (a_TRIS_) is 0.79 Bq. This correlates to a turnover of less than 1 millionth of the total radioactivity used. For a 10-min measurement time of the tested cocktails, this measurement is still detectable as the MDL (Eq. 4) lies between 0.59–0.63 Bq for the different cocktails (4). These values correspond to minimum detection limits of 0.075–0.08 pmol cm^−3^ day^−1^. A 60-min measurement time would reduce the MDL (4) of SRR to 0.065–0.071 pmol cm^−3^ d^−1^. These hypothetical values compare well to low turnover rate measurements with similar parameters in the literature that can be found in the range of approximately 0.1–10 pmol cm^−3^ d^−1^ (Beulig et al., 2022; Nagakura et al., 2022).

**Figure 5:**
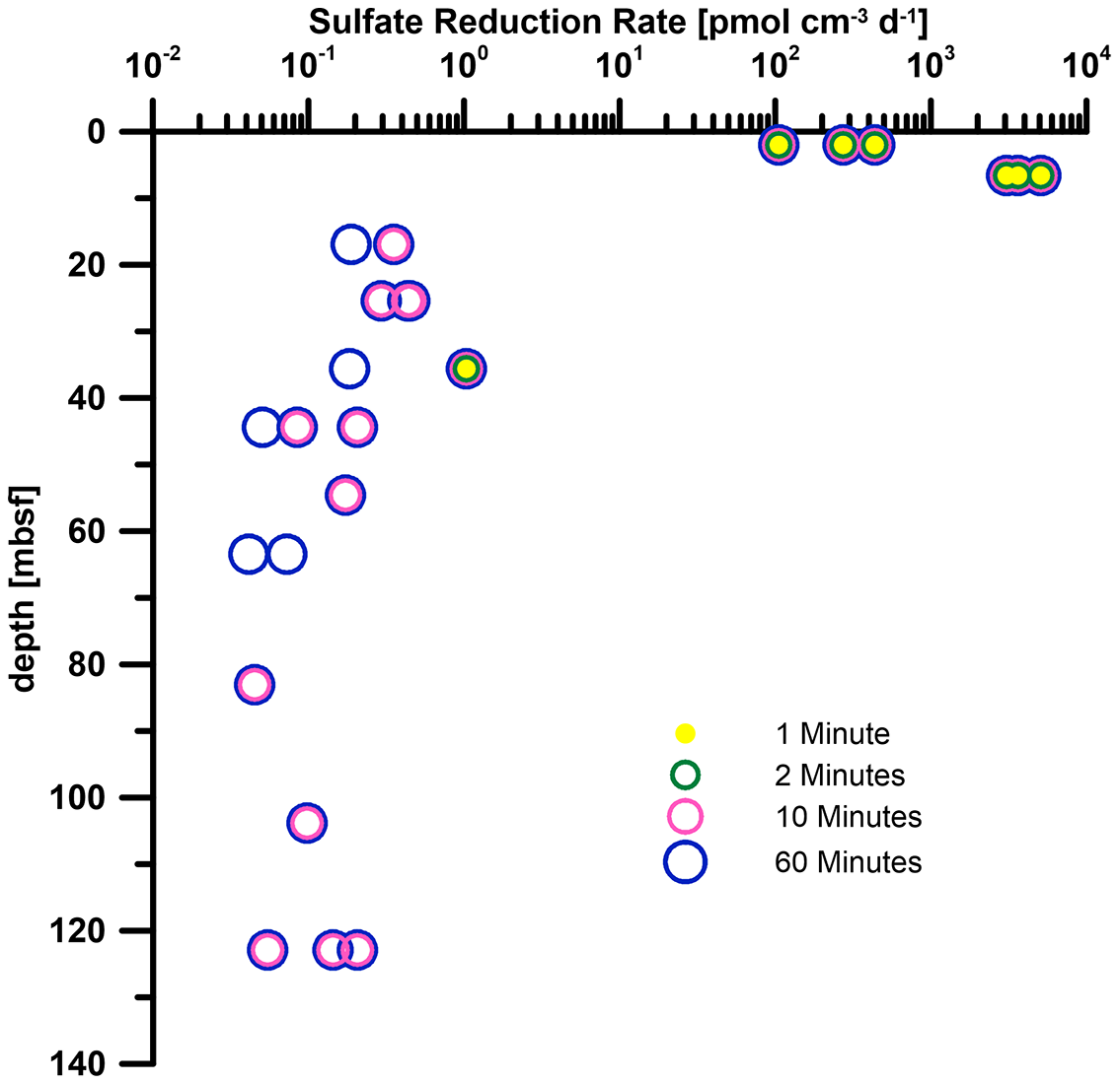
Sulfate reduction rates of IODP Exp. 385 Site U1545 (Nagakura et al., 2022), calculated by using standard deviations from different measurement times (Table 3). Measurements were carried out with AquaLight+ scintillation cocktail.

For methods that rely on measurements via gas solubility such as anaerobic oxidation of methane, these reductions of the minimum detection limit are especially important as the amount of radiotracer cannot be increased as freely as for other processes such as sulfate reduction or methanogenesis due to the constrains of dissolution of the tracer in the media (Beulig et al., 2022). We highly encourage the inclusion of the measurement time in the method description in all studies to allow for better comparability and reproducibility of data. Reduced standard deviation (Table 3, AquaLight+) can have significant impact on data (Figure 5) from the deep biosphere (Nagakura et al., 2022) as the margin of detection is slim for turnover rates near the limit of detection. The high variance in our 1- and 2-min measurements only covers SRR values above 0.1 pmol cm^−3^ day^−1^. Almost two more magnitudes of data (just slightly above 1 fmol cm^−3^ day^−1^) are detectable by switching from 1 and 2 min measurement time to 10 or 60 min with their reduced variance. As a result, turnover rates over the entire depth range can be observed instead of only the samples from the shallowest depth.

### Temperature

Temperature can have a strong influence on liquid scintillation counting as the scintillation process can be accelerated with increasing temperatures (Birks, 1964) and counting efficiency can decrease (Homma and Murase, 1987). Generally, conditions around 20°C are advised by the manufacturers of the scintillation cocktails. As for many other parameters, it is advisable to keep the LSC in a temperature-controlled environment with as little fluctuation as possible.

**Table 4:** Minimum detection limit of selected scintillation cocktails at various measurement times.

MDL [CPM3]	1 min	2 min	10 min	60 min
ROTISZINT	24.17	20.38	16.04	13,99
AquaLight+	24.20	19.08	16.48	14.97
Ultima Gold XR	119.06	13.94	10.53	9.16

The miminum detection limit is presented based on CPM3 of the counter background values (Table 2).

Our experiment shows that between 10 and 20°C, temperature does not influence the background measurements (Figure 3). At temperatures around the upper limit of the recommended operating temperatures, i.e., above *ca.* 25°C slightly elevated background measurements can be observed. While this increase will not be noticeable during most measurements, as the number of detected events it is still negligible compared to the events caused by decays of the radioisotope in the sample, it might already be critical for measurements close to the lower limit of detection. At temperatures of 28°C and higher, the counter background increases significantly to values that are almost an order of magnitude above the counter background at 20°C, hence on a level that precludes low turnover measurements due to the drastically increased detection limit.

We also investigated potential chemical alterations of the scintillation cocktail due to increased storage temperatures. While the measurement temperature had a significant effect on the background (Figure 3), the storage temperature only has a minimal to no effect (Figure 4). While samples of all storage temperatures are roughly spread around the 20°C control, measurements around 36°C storage temperature visibly deviate to up to 190% of the 20°C reference samples. This increase can be attributed solely to the measurements of the ROTI scintillation cocktail. The effect of storage temperature on the counter background still is minimal to negligible compared to the measurement temperature.

We repeated both the storage and measurement temperature experiments with a UG scintillation cocktail that was exposed to extended periods of intense heating due to sunlight. Compared to the same cocktail stored according to the manufacturer’s guidelines no significant difference was observed (data not shown).

## Conclusion

It is vital to understand the mechanisms and parameters surrounding liquid scintillation measurements, especially when working close to the minimum detection limit. Samples with low microbial activity are especially susceptible to minor variations within the counter background as these fluctuations are disproportionally affecting the detection limit. To avoid a negative impact of luminosity on measurements, samples should be stored prior to measurement at least 1 day and up to 4 days, depending on the scintillation cocktail. Both samples and the liquid scintillation analyzer should be kept at temperatures at around 20°C to avoid elevated counter background. While scintillation cocktails perform equally well under standard conditions (room temperature, appropriate measurement time), cocktails should be selected to meet the needs of specific experiments and parameters. At last, we showed that for low activity measurements such as the counter background or samples with low turnover, a measurement time of 10–60 min should be chosen. Information about counting times should be included in the respective method section of each manuscript to provide a more concise picture of the analytical conditions.

## Data availability statement

The raw data supporting the conclusions of this article will be made available by the authors, without undue reservation.

## Author contributions

FS and JK designed the study. FS conducted all experiments, and wrote the manuscript with input from JK. All authors contributed to the article and approved the submitted version.

## Funding

FS was funded through DFG grant (#651694) to JK.

## Conflict of interest

The authors declare that the research was conducted in the absence of any commercial or financial relationships that could be construed as a potential conflict of interest.

## Publisher’s note

All claims expressed in this article are solely those of the authors and do not necessarily represent those of their affiliated organizations, or those of the publisher, the editors and the reviewers. Any product that may be evaluated in this article, or claim that may be made by its manufacturer, is not guaranteed or endorsed by the publisher.

## References

[ref1] Ashley J. (1982). Stopping power of liquid water for low-energy electrons. Radiat. Res. 89, 25–31. doi: 10.2307/3575681

[ref2] Baker B. J. Moser D. P. MacGregor B. J. Fishbain S. Wagner M. Fry N. K.. (2003). Related assemblages of sulphate-reducing bacteria associated with ultradeep gold mines of South Africa and deep basalt aquifers of Washington state. Environ. Microbiol. 5, 267–277. doi: 10.1046/j.1462-2920.2003.00408.x, PMID: 12662174

[ref3] Bar-On Y. M. Phillips R. Milo R. (2018). The biomass distribution on earth. Proc. Natl. Acad. Sci. 115, 6506–6511. doi: 10.1073/pnas.1711842115, PMID: 29784790 PMC6016768

[ref4] Beulig F. Røy H. Glombitza C. Jørgensen B. (2018). Control on rate and pathway of anaerobic organic carbon degradation in the seabed. Proc. Natl. Acad. Sci. 115, 367–372. doi: 10.1073/pnas.1715789115, PMID: 29279408 PMC5777060

[ref5] Beulig F. Schubert F. Adhikari R. R. Glombitza C. Heuer V. B. Hinrichs K.-U.. (2022). Rapid metabolism fosters microbial survival in the deep, hot subseafloor biosphere. Nat. Commun. 13, 1–9. doi: 10.1038/s41467-021-27802-7 35078973 PMC8789916

[ref6] Birks J. (1964). The Theory and Practice of Scintillation Counting. Oxford, England: Pergamon Press

[ref7] Boetius A. Ravenschlag K. Schubert C. J. Rickert D. Widdel F. Gieseke A.. (2000). A marine microbial consortium apparently mediating anaerobic oxidation of methane. Nature 407, 623–626. doi: 10.1038/35036572, PMID: 11034209

[ref8] Böning P. Brumsack H.-J. Böttcher M. E. Schnetger B. Kriete C. Kallmeyer J.. (2004). Geochemistry of Peruvian near-surface sediments. Geochim. Cosmochim. Acta 68, 4429–4451. doi: 10.1016/j.gca.2004.04.027

[ref9] Broda R. (2003). A review of the triple-to-double coincidence ratio (TDCR) method for standardizing radionuclides. Appl. Radiat. Isot. 58, 585–594. doi: 10.1016/S0969-8043(03)00056-3, PMID: 12735976

[ref10] Broda R. Cassette P. Kossert K. (2007). Radionuclide metrology using liquid scintillation counting. Metrologia 44, S36–S52. doi: 10.1088/0026-1394/44/4/S06

[ref11] Broser I. Kallmann H. (1947). Über den Elementarprozeß der Lichtanregung in Leuchtstoffen durch α-Teilchen, schnelle Elektronen und γ-Quanten II. Zeitschrift für Naturforschung A 2, 642–650. doi: 10.1515/zna-1947-11-1206

[ref12] Brüchle W. (2003). Confidence intervals for experiments with background and small numbers of events. Radiochim. Acta 91, 71–80. doi: 10.1524/ract.91.2.71.19989

[ref13] Cassette P. Broda R. Hainos D. Terlikowska T. (2000). Analysis of detection-efficiency variation techniques for the implementation of the TDCR method in liquid scintillation counting. Appl. Radiat. Isot. 52, 643–648. doi: 10.1016/S0969-8043(99)00224-9, PMID: 10724420

[ref14] Čerenkov P. A. (1937). Visible radiation produced by electrons moving in a medium with velocities exceeding that of light. Phys. Rev. 52, 378–379. doi: 10.1103/PhysRev.52.378

[ref15] Cowan D. A. (2004). The upper temperature for life–where do we draw the line? Trends Microbiol. 12, 58–60. doi: 10.1016/j.tim.2003.12.002, PMID: 15040324

[ref16] Csala M. Senesi S. Bánhegyi G. Mandl J. Benedetti A. (2005). Characterization of sulfate transport in the hepatic endoplasmic reticulum. Arch. Biochem. Biophys. 440, 173–180. doi: 10.1016/j.abb.2005.06.017, PMID: 16055076

[ref17] Fossing H. (1995). 35S-Radiolabeling to Probe Biogeochemical Cycling of Sulfur. Washington, DC: ACS Publications.

[ref18] Glombitza C. Adhikari R. R. Riedinger N. Gilhooly W. P. III Hinrichs K.-U. Inagaki F. (2016). Microbial sulfate reduction potential in coal-bearing sediments down to~ 2.5 km below the seafloor off Shimokita peninsula, Japan. Front. Microbiol. 7:1576. doi: 10.3389/fmicb.2016.01576, PMID: 27761134 PMC5051215

[ref19] Herforth L., (1948). Die Fluoreszenzanregung Organischer Substanzen Mit Alphateilchen, Schnellen Elektronen und Gammastrahlen. Berlin: Technische Universität

[ref20] Heuer V. B. Inagaki F. Morono Y. Kubo Y. Spivack A. J. Viehweger B.. (2020). Temperature limits to deep subseafloor life in the Nankai trough subduction zone. Science 370, 1230–1234. doi: 10.1126/science.abd7934, PMID: 33273103

[ref21] Hinrichs K.-U. Inagaki F. (2012). Downsizing the deep biosphere. Science 338, 204–205. doi: 10.1126/science.1229296, PMID: 23066067

[ref22] Homma Y. Murase Y. (1987). Energy resolution of liquid scintillator for α-particles and internal conversion electrons at lower temperatures. J. Radioanal. Nucl. Chem. 119, 355–365. doi: 10.1007/BF02162046

[ref23] Horrocks D. L. (1974). Applications of Liquid Scintillation. Academic Press, New York and London: Citeseer.

[ref24] Horrocks D. L. (1985). Studies of background sources in liquid scintillation counting. Int. J. Appl. Radiat. Isot. 36, 609–617. doi: 10.1016/0020-708X(85)90001-8

[ref25] Horrocks D. (2012). Applications of Liquid Scintillation Counting. Cambridge, Massachusetts, USA: Elsevier.

[ref26] Inagaki F. Hinrichs K.-U. Kubo Y. Bowles M. W. Heuer V. B. Hong W.-L.. (2015). Exploring deep microbial life in coal-bearing sediment down to ~2.5 km below the ocean floor. Science 349, 420–424. doi: 10.1126/science.aaa6882, PMID: 26206933

[ref27] Ivanov M. V. (1956). Isotopes in the determination of the sulfate-reduction rate in Lake Belovod. Microbiologiya 25, 305–309.

[ref28] Iversen N. Blackburn T. H. (1981). Seasonal rates of methane oxidation in anoxic marine sediments. Appl. Environ. Microbiol. 41, 1295–1300. doi: 10.1128/aem.41.6.1295-1300.1981, PMID: 16345784 PMC243914

[ref29] Jørgensen B. B. (1982). Mineralization of organic matter in the sea bed—the role of sulphate reduction. Nature 296, 643–645. doi: 10.1038/296643a0

[ref30] Jørgensen B. B. (2021). Sulfur biogeochemical cycle of marine sediments. Geochem. Perspect. 10, 145–307. doi: 10.7185/geochempersp.10.2

[ref31] Jørgensen B. B. Boetius A. (2007). Feast and famine—microbial life in the deep-sea bed. Nat. Rev. Microbiol. 5, 770–781. doi: 10.1038/nrmicro1745, PMID: 17828281

[ref32] Jørgensen B. Fenchel T. (1974). The sulfur cycle of a marine sediment model system. Mar. Biol. 24, 189–201. doi: 10.1007/BF00391893

[ref33] Jørgensen B. B. Findlay A. J. Pellerin A. (2019). The biogeochemical sulfur cycle of marine sediments. Front. Microbiol. 10:849. doi: 10.3389/fmicb.2019.00849, PMID: 31105660 PMC6492693

[ref34] Kaihola L. Ross H. Noakes J. Spaulding J., (1991). Liquid Scintillation Counting Performance Using Glass Vials in the Wallac 1220 Quantulus. Liquid Scintillation Counting and Organic Scintillators. Chelsea: Lewis Publishers. 495–500

[ref35] Kaiser H. (1970). Report for analytical chemists. II. Quantitation in elemental analysis. Lab. Guide Instrum. Equip. Chem. 42, 26A–59A. doi: 10.1021/ac60286a027

[ref36] Kallmann H. (1950). Scintillation counting with solutions. Phys. Rev. D. Partic. Fields 78, 621–622. doi: 10.1103/PhysRev.78.621.2

[ref37] Kallmeyer J. Boetius A. (2004). Effects of temperature and pressure on sulfate reduction and anaerobic oxidation of methane in hydrothermal sediments of Guaymas Basin. Appl. Environ. Microbiol. 70, 1231–1233. doi: 10.1128/AEM.70.2.1231-1233.2004, PMID: 14766611 PMC348843

[ref38] Kallmeyer J. Ferdelman T. G. Weber A. Fossing H. Jørgensen B. B. (2004). A cold chromium distillation procedure for radiolabeled sulfide applied to sulfate reduction measurements. Limnol. Oceanogr. Methods 2, 171–180. doi: 10.4319/lom.2004.2.171

[ref39] Kallmeyer J. Pockalny R. Adhikari R. R. Smith D. C. D’Hondt S. (2012). Global distribution of microbial abundance and biomass in subseafloor sediment. Proc. Natl. Acad. Sci. 109, 16213–16216. doi: 10.1073/pnas.1203849109, PMID: 22927371 PMC3479597

[ref40] Kobayashi Y. (2012). Biological Applications of Liquid Scintillation Counting. Cambridge, Massachusetts, USA: Elsevier.

[ref41] Kojola H. Polach H. Nurmi J. Oikari T. Soini E. (1984). High resolution low-level liquid scintillation β-spectrometer. Int. J. Appl. Radiat. Isot. 35, 949–952. doi: 10.1016/0020-708X(84)90208-4

[ref42] Kossert K. (2010). Activity standardization by means of a new TDCR-Čerenkov counting technique. Appl. Radiat. Isot. 68, 1116–1120. doi: 10.1016/j.apradiso.2009.12.038, PMID: 20096596

[ref43] Krebs A. T. (1955). Early history of the scintillation counter. Science 122, 17–18. doi: 10.1126/science.122.3157.17, PMID: 14385812

[ref44] L’Annunziata M. F. (2020). Handbook of Radioactivity Analysis, 2 Cambridge, Massachusetts, USA: Radioanalytical Applications. Academic Press.

[ref45] L’Annunziata M. F. Tarancón A. Bagán H. García J. F. (2020). Liquid Scintillation Analysis: Principles and Practice, Handbook of Radioactivity Analysis Cambridge, Massachusetts, USA: Elsevier, 575–801.

[ref46] Magnabosco C. Lin L.-H. Dong H. Bomberg M. Ghiorse W. Stan-Lotter H.. (2018). The biomass and biodiversity of the continental subsurface. Nat. Geosci. 11, 707–717. doi: 10.1038/s41561-018-0221-6

[ref47] Meyer S. Schweidler E. Meyer S. Schweidler E. (1927). Die Radioaktiven Substanzen. Wiesbaden, Germany: Springer.

[ref48] Morono Y. Inagaki F. (2016). Analysis of low-biomass microbial communities in the deep biosphere. Adv. Appl. Microbiol. 95, 149–178. doi: 10.1016/bs.aambs.2016.04.001, PMID: 27261783

[ref49] Morono Y. Terada T. Kallmeyer J. Inagaki F. (2013). An improved cell separation technique for marine subsurface sediments: applications for high-throughput analysis using flow cytometry and cell sorting. Environ. Microbiol. 15, 2841–2849. doi: 10.1111/1462-2920.12153, PMID: 23731283 PMC3910163

[ref50] Nagakura T. Schubert F. Wagner D. Kallmeyer J. Party S. S. (2022). Biological sulfate reduction in deep subseafloor sediment of Guaymas Basin. Front. Microbiol. 462 doi: 10.3389/fmicb.2022.845250 PMC8927301 35308366

[ref51] Parkes R. J. Cragg B. A. Bale S. Getlifff J. Goodman K. Rochelle P. A.. (1994). Deep bacterial biosphere in Pacific Ocean sediments. Nature 371, 410–413. doi: 10.1038/371410a0

[ref52] Reynolds G. T. Harrison F. B. Salvini G. (1950). Liquid scintillation counters. Phys. Rev. D. Partic. Fields 78:488. doi: 10.1103/PhysRev.78.488

[ref53] Røy H. Weber H. S. Tarpgaard I. H. Ferdelman T. G. Jørgensen B. B. (2014). Determination of dissimilatory sulfate reduction rates in marine sediment via radioactive 35S tracer. Limnol. Oceanogr. Methods 12, 196–211. doi: 10.4319/lom.2014.12.196

[ref54] Shock E. (2000). Characterizing the biotic fringe in hydrothermal ecosystems. Goldschmidt Conference, Oxford, UK J. Conf. Abstr.:923.

[ref55] Simpson B. Meyer B. (1994). Direct activity measurement of pure beta-emitting radionuclides by the TDCR efficiency calculation technique. Nucl. Instrum. Methods Phys. Res. Sect. A 339, 14–20. doi: 10.1016/0168-9002(94)91771-X

[ref56] Smith R. L. Klug M. J. (1981). Electron donors utilized by sulfate-reducing bacteria in eutrophic lake sediments. Appl. Environ. Microbiol. 42, 116–121. doi: 10.1128/aem.42.1.116-121.1981, PMID: 16345804 PMC243972

[ref57] Sorokin Y. I. (1962). Experimental investigation of bacterial sulfate reduction in the Black Sea using S 35. Microbiology 31, 329–335.

[ref58] Temple S. (2015). Liquid scintillation counting: how has it advanced over the years and what does the future hold? Bioanalysis 7, 503–505. doi: 10.4155/bio.15.10, PMID: 25826131

[ref59] Treude T. Niggemann J. Kallmeyer J. Wintersteller P. Schubert C. J. Boetius A.. (2005). Anaerobic oxidation of methane and sulfate reduction along the Chilean continental margin. Geochim. Cosmochim. Acta 69, 2767–2779. doi: 10.1016/j.gca.2005.01.002

